# Presence and leaching of bisphenol a (BPA) from dental materials

**DOI:** 10.1080/23337931.2018.1476869

**Published:** 2018-05-27

**Authors:** Rune Becher, Hanne Wellendorf, Amrit Kaur Sakhi, Jan Tore Samuelsen, Cathrine Thomsen, Anette Kocbach Bølling, Hilde Molvig Kopperud

**Affiliations:** a Nordic Institute of Dental Materials (NIOM), Oslo, Norway;; b Norwegian Institute of Public Health, Oslo, Norway

**Keywords:** Dental materials, BPA leaching, health impact

## Abstract

BPA has been reported to leach from some resin based dental restorative materials and materials used for orthodontic treatment. To confirm and update previous findings, especially in light of the new temporary lower threshold value for tolerable daily BPA intake, we have investigated the leaching of BPA from 4 composite filling materials, 3 sealants and 2 orthodontic bonding materials. The materials were either uncured and dissolved in methanol or cured. The cured materials were kept in deionized water for 24 hours or 2 weeks. Samples were subsequently analyzed by ultra-performance liquid chromatography coupled to mass spectrometry (UPLC-MS-MS). The composite filling material Tetric EvoFlow^®^ and the fissure sealant DELTON^®^ showed significantly higher levels of BPA leaching compared to control samples for all test conditions (uncured, 24 h leaching and 2 weeks leaching). There were no significant differences in amount of leached BPA for any of the tested materials after 24 hours compared to 2 weeks. These results show that BPA is still released from some dental materials despite the general concern about potential adverse effects of BPA. However, the amounts of BPA were relatively low and most likely represent a very small contribution to the total BPA exposure.

## Introduction

Bisphenol A (BPA) is a high production volume chemical mainly used for manufacturing polycarbonate plastics, epoxy resins and methacrylate resins used in dentistry. In animal studies, a number of adverse health effects have been associated with BPA including effects on hormonal activity, asthma, diabetes, obesity, behavioral changes, cancer, infertility and genital malformations [1–6]. The largest source for human exposure is food packed in BPA-containing materials. A recent review by EFSA (the European Organization for Food Safety) concluded that rats and mice exposed to BPA are likely to experience effects on the general health of liver and kidneys as well as on breast tissue in terms of increased cell growth [7]. Since the most serious effect at the lowest BPA concentration was observed in kidneys, increased focus on the kidneys of mice was used as the basis for a new threshold value for tolerable daily intake (TDI). In mice, adverse effects on kidney are observed at about 600 micrograms per kg body weight per day. The new TDI value was lowered from 50 to 4 µg per kg body weight per day, using an uncertainty factor of 150 [7]. Due to uncertainty about possible effects on breast, reproductive, neurological and metabolic systems, as well as the immune system in laboratory animals, the new TDI value is temporary and a new evaluation of BPA will be performed by EFSA in near future.

Effects of BPA in humans are debated, but it is worth noting that in a cross-sectional study of 1,500 individuals published in 2008, BPA exposure was associated with heart disease and diabetes [8]. These results were further supported in a later study [9]. BPA in a dental context has often been linked to fissure sealants and composite materials where its presence is likely due to an impurity from the manufacturing process [10]. Increased concentrations of BPA in saliva and urine have been demonstrated after use of such materials in dental treatment [11–15]. However, other materials may not give BPA leaching [16], indicating that the leaching of BPA is material dependent.

A study from 2012 found that children who had received composite fillings had more behavioral problems than the control group [17]. The authors suggested this to be due to a possible BPA content in the materials. However, there were no significant differences in physical development over five years for children with either amalgam or composite fillings [18]. It is too early to say whether such studies show actual causal or random correlations. A systematic review by the Norwegian Institute of Public Health (NIPH) of any adverse health effects from composite materials found that very few studies were done with sufficient quality to make a good assessment. The available evidence was rated as moderate to low quality, but it did not appear that it played a “role whether children received amalgam fillings or composite fillings, when it came to a variety of outcomes related to health” [19].

SCENIHR (Scientific Committee on Emerging and Newly Identified Health Risks) recently published a report on the safety of BPA in medical devices, including dental materials [20]. The report concluded that both short- and long-term exposure of BPA from dental materials are below the temporary TDI value of 4 µg/kg bw/day. For children, exposure was estimated to be 140 and 2 ng/kg bw/day from dental materials for short and long term exposure respectively. SCENIHR also concluded that there is some uncertainty associated with many of the reported values of BPA leaching from dental materials. In line with this, a recent study showed confounding results as to the whether the detected substances were actually BPA [21]. These differences appear to be particularly related to the method of analysis. Here, we have used an analysis method in which the possibility of interferences is small.

The aim of this study was (a) to find out whether resin-based dental materials in uncured form contain bisphenol A (BPA), and (b) to study whether BPA leached from these dental materials the first 24 h after curing and 2 weeks after curing. For this purpose a selection of commonly used materials (restorative materials, fissure-sealants and bonding agents) were used. Furthermore, we wanted to estimate the BPA exposure through dental sealants and composites in both adults and children.

## Materials and methods

Dental materials containing monomers bis-GMA or bis-EMA as potential source of BPA were selected for the study. All test materials were from the manufacturers (see supplemental data, Table S1). These materials included four composite restorative materials, three fissure-sealants and two bonding agents used in orthodontic treatment (Table 1). Both BPA and its C-13 labeled internal standard were bought from Cambridge Isotope Laboratories Inc. (Andover, MA, USA).

**Table 1.** Materials tested.

Composites	Fissure sealants	Orthodontic bonding
Ceram.X^®^ (Dentsply DeTrey GmbH)	Clinpro™ Sealant (3M ESPE)	Transbond™PLUS (3M Unitek Orthodontic Products)
Grandio^®^ Flow (VOCO GmbH)	DELTON^®^ (Dentsply DeTrey GmbH)	Band-Lok^®^ (Reliance Orthodontic Products Inc. Part A og Part B)
Filtek™ Supreme XTE (3M ESPE)	Helioseal^®^ F (Ivoclar Vivadent AG)	
Tetric EvoFlow^®^ (Ivoclar Vivadent AG)		

### Study design

Two separate studies were performed. Initially, we conducted a pilot study (study 1) where fewer numbers (*n* = 3) of parallels were analyzed. In study 1, hydroquinone was added to all samples, including controls, as a mean to stabilize leached material. BPA levels in uncured material and BPA leached after 24 h and 2 weeks were determined. The following study 2 had no hydroquinone added, higher numbers of parallels (*n* = 5) and BPA leaching was measured after 24 h only.

### Uncured materials

Uncured material was weighed (about 25–50 mg) and dissolved in methanol (5 ml). One of the bonding materials (Band-Lok^®^) consisted of two components. Both components were analyzed individually in uncured form. Triplicate samples of each material were prepared. After dissolution, the solutions were centrifuged to remove the filler and undissolved compounds. The supernatant was stored at −18 °C until assayed.

### Cured materials

ISO 10993–12 was used as a guideline for the ratio between the test material's total surface area and extraction volume, recommended to 3 cm^2^/ml [22]. Due to the materials' different applications (filling, sealing, bonding) test bodies of different size and number were used for the different types of material, but with equal extraction volume in relation to surface area. The curing light bluephase 20i (Ivoclar Vivadent, Schaan, Liechtenstein) was used in High Power mode at the top surface of the specimens. The curing times were according to the individual material manufacturers’ recommendations.

Specimens of each material were placed in glass vials directly after curing for assessment of leaching. In the first part of the study (Study 1, comparison of leaching after 24 h and 2 weeks), the leaching medium was deionized water with the addition of hydroquinone (HQ) for stabilizing any leached material. Sample vials were kept in shaking water bath (100 rpm) at 37 °C for 24 h and 14 days, respectively. After extraction, the solutions were transferred to the assay vial and stored at −18 °C until analyzed. The same procedure was used in study 2 (leaching after 24 h) with the exception that HQ was not added to the leaching medium.

For composites, the prepared test specimens each had a diameter of 5 mm and a height of 2 mm in both study 1 and 2. The total surface area per analysis of the composites was thus 141.4 mm^2^ (2 specimens). For the fissure sealants and the orthodontic bonding materials, the test specimens also had a diameter of 5 mm. In the first study the thickness of fissure sealants and the orthodontic bonding material specimens were 1 mm and in the second study 0.5 mm. The total surface area per analysis of fissure sealants and the orthodontic bonding materials was thus 164.9 mm^2^ in study 1 (3 Specimens) and 141.4 mm^2^ in study 2 (3 specimens). All sample volumes were 0.5 ml. The amounts in nanograms per square mm were calculated by dividing the absolute amount leached BPA with the surface area of the test specimen.

### Method of analysis

The analyses were conducted at the NIPH as previously described [23]. In brief, C-13 labeled BPA internal standard was added to all samples and analyzed using ultra performance liquid chromatography coupled to mass spectrometry (UPLC-MS-MS). Blank and control samples were analyzed simultaneously with the samples. The limit of detection (LOD) and limit of quantification (LOQ) were 0.04 ng/ml and 0.1 ng/ml respectively. The relative standard deviation for in-house controls (*n* = 187) and standard reference material SRM 3673 (*n* = 77) from National Institute of Standards and Technology (NIST) was 26% (23). In addition, we have participated in two inter-laboratory comparisons (External Quality Assessment Scheme for Organic Substances in Urine (OSEQAS) and German External Quality Assessment Scheme (G-EQUAS)). The z-scores were less than 1.5 and the concentrations were within tolerance range [23].

### Statistics

For both uncured and cured materials in study 1, the data were log transformed before performing ANOVA to fulfill the assumption of equal standard deviations of all sets of replicates.

For the uncured materials, the presence of statistically significant values above control was evaluated by one-way ANOVA followed by Dunnett’s multiple comparison tests. For the cured materials in study 1 with two leaching times, statistical significances between control and treatment groups and between length of leaching time (24 h and 2 weeks) were evaluated by two-way ANOVA. There was no interaction between the dental material factor and the time factor, allowing for post-test (Bonferroni’s multiple comparison test) within the dental material factor.

Log transformation was not performed on the data for the cured materials in study 2 (only 24 h leaching time), since some of the control values were 0, resulting in no value after the transformation. Statistical significance was thus evaluated on the raw data using one-way ANOVA with Dunnett’s multiple comparison test.

All analyses were performed using the statistical software GRAPHPAD PRISM (GraphPad Software, San Diego, CA, USA). Results were calculated as mean ± SD and *p* values <.05 was considered statistically significant.

## Results

### Study 1

#### Control samples

The control samples in study 1 consisted of water with added hydroquinone in the same amount as the material samples received. The controls for the uncured samples had a mean BPA level of 2.7 ± 0.2 ng/ml, whereas controls for 24 h and 2 weeks had background levels of 1.7 ± 0.2 and 1.6 ± 0.4 ng/ml respectively ( Tables 2–4).

**Table 2.** Study 1. Presence of BPA in uncured materials. Only materials with leaching significantly above control are shown.

Material	Sample number	BPA leaching Mean ± SD [ng/ml]	BPA in thousands of sample weight Mean ± SD [0/00]
Control	2	2.7 ± 0.2	n.a
Tetric EvoFlow^®^	3	22 ± 3	0.005 ± 0.001
DELTON^®^	3	87 ± 26	0.013 ± 0.001

**Table 3.** Study 1. Total leaching of BPA from cured materials after immersion in deionized water for 24 h. Only materials with leaching significantly above controls are shown.

Material	Sample number	BPA leaching Mean ± SD [ng/ml]	BPA leaching Mean ± SD [ng/cm^2^]
Control	3	1.7 ± 0.2	n. a.
Tetric EvoFlow^®^	3	7.3 ± 1.3	2.6 ± 0.5
DELTON^®^	3	6.2 ± 0.3	1.9 ± 0.1

**Table 4.** Study 1. Total leaching of BPA from cured materials after immersion in deionized water for 2 weeks. Only materials with leaching significantly above controls are shown.

Material	Sample number	BPA leaching Mean ± SD [ng/ml]	BPA leaching Mean ± SD [ng/cm^2^]
Control	3	1.6 ± 0.4	n. a.
Tetric EvoFlow^®^	3	8.7 ± 1.6.	3.1 ± 0.6
DELTON^®^	3	9.2 ± 2.1	2.8 ± 0.7

#### BPA in uncured materials

Among the 4 composites, only Tetric EvoFlow^®^ contained BPA levels significantly above the control levels (mean 22 ± 3 ng/ml) whereas DELTON^®^ was the only fissure sealant with BPA levels significantly above the control levels (mean 87 ± 26 ng/ml) (Table 2). None of the uncured bonding materials contained levels of BPA significantly above the control values (data not shown).

#### Cured materials, BPA leaching 24h

Tetric EvoFlow^®^ was the only cured composite and DELTON^®^ the only cured fissure sealant that leached BPA levels significantly above the controls with mean values of 7.3 ± 1.3 ng/ml (2.6 ± 0.5 ng/cm^2^) and 6.2 ± 0.3 ng/ml (1.9 ± 0.1 ng/cm^2^), respectively (Table 3). None of the cured bonding materials showed significantly higher leaching of BPA compared with the control values (data shown in Supplement, Table S2).

#### Cured materials, BPA leaching 2 weeks

Similar to the results of 24 h leaching, Tetric EvoFlow^®^ and DELTON^®^ were the only cured materials leaching BPA significantly above the controls with means of 8.7 ± 1.6 ng/ml (3.1 ± 0.6 ng/cm^2^) and 9.2 ± 2.2 ng/ml (2.8 ± 0.7 ng/cm^2^), respectively (Table 4). None of the bonding materials had amounts of leached BPA levels significantly different from the controls (data shown in Supplement, Table S3).

**Figure 1.**
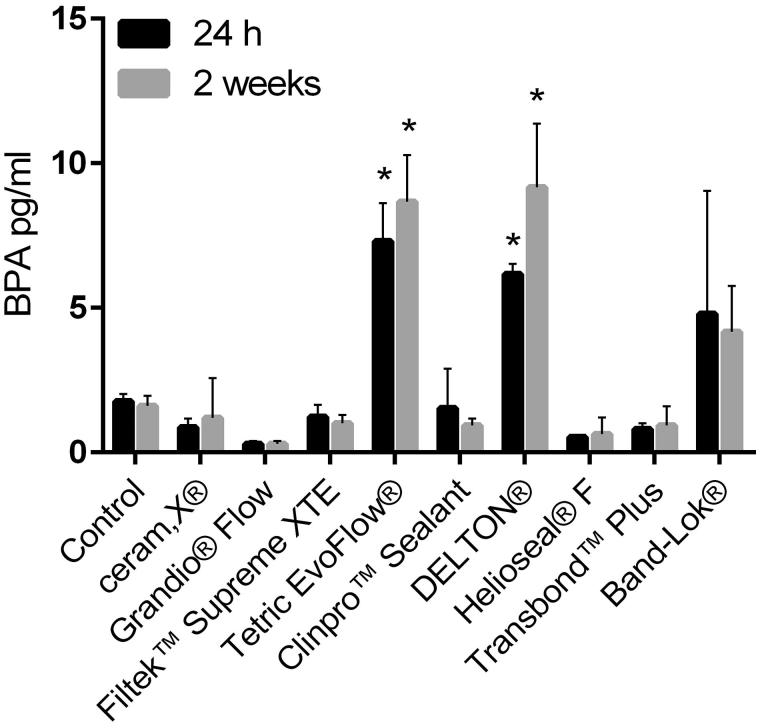
Study 1. Total leaching of BPA from cured materials after immersion in deionized water for 24 h and 2 weeks (*n* = 3). Bars are shown as mean ± SD. *p* values <.05 was considered statistically significant. In the two-way ANOVA, there was a significant effect of the dental material factor, but not for the time factor. Moreover, there was no interaction between the two factors, allowing for Bonferroni post-tests within the dental material factor. *indicates significantly different from control at the same leaching time.

For the tested materials, there were no statistically significant differences between the leached amounts of BPA at 24 h and 2 weeks as visualized in Figure 1.

### Study 2

#### Control samples

The control samples in study 2 consisted of deionized water without hydroquinone. The results showed that the control samples had no detectable amounts of BPA (Table 5) (For complete data set, see supplemental data Table S4).

**Table 5.** Study 2. Total leaching of BPA from cured materials after immersion in deionized water for 24 h.

Material	Sample number	BPA leaching Mean ± SD [ng/ml]	BPA leaching Mean ± SD [ng/cm^2^]
Control (Blank)	3	0.00 ± 0.01	n.a.
ceram.X^®^	5	0.36 ± 0.06	0.13 ± 0.02
Grandio^®^ Flow	5	0.08 ± 0.01	0.029 ± 0.005
Filtek™ Supreme XTE	5	0.6 ± 0.1	0.20 ± 0.04
Tetric EvoFlow^®^	5	6.5 ± 0.4^a^	2.3 ± 0.2^a^
Clinpro™ Sealant	5	0.17 ± 0.09	0.06 ± 0.03
DELTON^®^	5	9.6 ± 2.2^a^	3.4 ± 0.8^a^
Helioseal^®^ F	5	0.04 ± 0.04	0.01 ± 0.01
Transbond™ PLUS	5	0.09 ± 0.02	0.033 ± 0.006
Band-Lok^®^	5	1.5 ± 0.6	0.5 ± 0.2

a indicates significantly different from control.

#### Cured materials, BPA leaching 24 h

In agreement with the findings from the first study, Tetric EvoFlow^®^ and DELTON^®^ were the two cured materials with the highest BPA leaching with mean levels of 6.5 ± 0.4 and 9.6 ± 2.2 ng/ml respectively (2.3 ± 0.2 ng/cm^2^ and 3.4 ± 0.7 ng/cm^2^) which were both significantly above controls (Table 5). None of the other materials showed significant values of BPA-leaching above controls.

## Discussion

Bisphenol A is found in a wide variety of different consumer products including some dental materials. Here we present data from two sets of analyses of various composites, fissure sealants and bonding agents used in both Norwegian and international dental practices. Initially, we conducted a pilot study (study 1) where fewer numbers of parallels were analyzed. Here hydroquinone was added to all samples, including controls, as a mean to stabilize leached material. From a clinical perspective it is relevant to convert the amount of BPA leaching to the surface area of the material, as this would give a measure of how much of the material that is exposed to the oral environment in a patient. Thus, all leached amounts are given both as amount per ml and amount per surface area.

The results from study 1 showed BPA background levels in the controls. Thus, for most of the tested materials in study 1, both uncured and cured with leaching for 24 h or 2 weeks, the measured concentration of BPA in the extraction medium was not significantly different from the control values. The exceptions were consistently Tetric EvoFlow^®^ and DELTON^®^.

Comparing the leached BPA values obtained at 24 h and 2 weeks showed these to be in the same range with no statistical difference between any of the materials (Figure 1). This suggests that the leaching was highest the first 24 h after curing and that only minor amounts of BPA continued to leach onwards. This is in agreement with previous clinical results showing that the leaching of BPA was detectable only initially, and thereafter rapidly declined [12]. Based on this, we used only 24 h sample time in study 2. Moreover, hydroquinone was not added to any of the samples and controls in study 2 since it was suspected to be a source for the contamination observed in the controls of study 1. The lack of BPA in the control samples of study 2 confirmed hydroquinone as the most likely culprit for these BPA background levels.

The results in study 2 confirmed our findings in study 1 regarding Tetric EvoFlow^®^ and DELTON^®^ as the materials with the highest leaching of BPA. Still, in contrast to study 1, some materials (the composites Ceram.X^®^ and Filtek™ Supreme XTE) showed somewhat higher levels of BPA compared to the other materials, but this difference was not statistically significant. It is possible that removal of the hydroquinone as the source of BPA contamination made small amounts of BPA de facto leaching from the materials more easily detectable in study 2. The overall finding of some minute BPA leaching from all the investigated materials may be due to an impurity from the manufacturing of the monomers bis-GMA and bis-EMA. All the materials tested in the present study contained one or both of these monomers and BPA is used as a starting point for their production.

Overall, our results are in agreement with previous studies in that small and varying amounts of BPA are released from some dental materials. However, the contribution to the total BPA exposure and to potential adverse health effects is most likely negligible. EFSA recently estimated the contribution from dental sealants to be limited to 0.001% compared to total BPA exposure from all sources. This amount is clearly below safe intake limits set by government bodies worldwide. This is also consistent with the expression of the American Dental Association (ADA); there is no reason for concern for BPA exposure from dental materials of today. Similar conclusions were also made by SCENIHR [20] as exposure to BPA from dental materials was found to be below the new temporary t-TDI of 4 µg per kg body weight [7].

ADA Science Institute recently analyzed 12 dental sealants and concluded that they showed extremely low BPA release with a median amount of BPA released of 0.09 nanograms from the amount of sealant applied to four teeth [24]. Kingsman and coworkers measured BPA concentrations in the saliva of individuals before and up to 30 h after placement of a composite resin restoration [12]. The geometric mean of BPA in saliva above baseline within the first hour of placement was 0.21 ng/ml. After the first hour BPA was not detected in saliva at levels above baseline, indicating the BPA exposure from the composite to be brief and not persisting in saliva in detectable amounts after 60 min.

We do not have data from saliva, however if we assume that 0.034 ng/mm^2^ which is the highest mean BPA level we measured in the deionized water after 24 h, is what an individual may be exposed to over this time span, we can compare total amount with ADA [24] and per surface area with Van Landuyt [25]. The latter found a geometric mean BPA release of 0.01752 nmol/mm^2^. Converted to amount per surface area, this equals approximately 4.1 ng/mm^2^ (see supplemental data for calculation).

If we estimate the surface of the sealer used per tooth to cover an area of approximately 6 mm^2^ (2 mm × 3 mm) and the sealer is used on 4 primary molars, the total surface area is 24 mm^2^. With a leaching of 0.034 ng/mm^2^ from 4 teeth, the exposure will then be 0.82 ng the first 24 h which is higher than the 0.09 ng reported by ADA [24] but the leaching per surface area (mm^2^) is lower than the geometric mean reported by Van Landuyt [25]. However, the confidence interval of BPA levels reported in the work of Van Landuyt is large and our results are within the range of these levels.

For a child weighing 25 kilo, our estimate gives a daily dose of approximately 0.032 ng/kg bodyweight per day (bw/day). Compared to the t-TDI for BPA of 4000 ng/kg bw/day, the contribution from the sealant is far below this. It is also clearly lower than the estimated levels by SCENIHR of 140 and 2 ng/kg bw/day from dental materials for less than 24 h exposure and long term exposure, respectively. In comparison, the estimated average daily dietary intake of BPA for infants and toddlers older than 6 months is 375 ng/kg bw/day whereas for teenagers, adults and the older individuals the estimated average values range from 116 to 159 ng/kg bw/day. Also considering that the exposure to the low concentrations of BPA from the sealant will be brief, the contribution of BPA from sealant to the total exposure is negligible. Thus, the margin of safety is several orders of magnitude lower than either exposure limit. Trace levels of BPA from dental resins do not appear to present a health hazard based on current exposure limits, especially when one considers that the exposure predominantly is acute only during the first hour post-treatment. However, this is based largely on an assessment of the concentrations where toxic effects of BPA are observed. Low dose issues and possible estrogenic effects remain unclear and are therefore not considered here.

In conclusion, our results support previous studies in that small and varying amounts of BPA are released from dental materials containing bis-GMA. However, the contributions to the total BPA exposure and to potential adverse health effects are most likely negligible and shadowed by the beneficial role these materials have in oral treatment procedures.

## Supplementary Material

Supplemental Material
