# Supplementary material for: Presence and leaching of bisphenol a (BPA) from dental materials
Source: Acta Biomater Odontol Scand. 2018 May 27;4(1):56–62. doi: 10.1080/23337931.2018.1476869 (PMC5974758; doi:10.1080/23337931.2018.1476869)
Supplement: Supplemental Material [file IABO_A_1476869_SM5170.docx]

**Supplemental data**

**Table S1.** Tested materials and contents as given in manufacturers’ instruction and/or Safety Data Sheet (SDS)

| **Product** | **Content** |
| --- | --- |
| **Composites** | |
| **ceram.X®** | Dimethacrylate resins SDS: TEGDMA, Urethan modifisert bis-GMA, bis-EMA |
| **Grandio® Flow** | bis-GMA, TEGDMA, HEDMA, BHT |
| **Filtek™ Supreme XTE** | bis-GMA, UDMA, TEGDMA, PEGDMA, bis-EMA, BHT |
| **Tetric EvoFlow®** | Dimethacrylates/ SDS: bis-GMA, 1,10-decandiol dimethacrylate, UDMA |
| **Fissure sealants** | |
| **Clinpro™ Sealant** | bis-GMA, TEGDMA, HQ, Ethyl 4-dimethyl aminobenzoate, Diphenyliodonium hexafluorophosphate |
| **DELTON®** | Aromatic and aliphtic monomers SDS: TEGDMA, bis-GMA |
| **Helioseal® F** | bis-GMA, UDMA, TEGDMA |
| **Orthodontic bonding** | |
| **Transbond^TM^ PLUS** | PEG, bis-GMA, DPIHFP (Diphenyliodonium hexafluorophosphate) |
| **Band-Lok®** | Part A: HEMA, bis-GMA Part B: TEGDMA, bis-GMA |

**Table S2. Study 1.** Total BPA leaching from cured materials after 24 hours. * indicates significantly different from control.

| **Sample** | **BPA leaching [ng/ml]** | **BPA leaching [ng/cm^2^]** |
| --- | --- | --- |
| **Control** | **2.0** | **not appropriate** |
|  | **1.8** |  |
|  | **1.5** |  |
| Mean ± SD | **1.7 ± 0.23** |  |
| **ceram.X®** | **0.6** | **0.2** |
|  | **0.8** | **0.3** |
|  | **1.2** | **0.4** |
| Mean ± SD | **0.9 ± 0.31** | **0.3 ± 0.11** |
| **Grandio® Flow** | **0.2** | **0.1** |
|  | **0.3** | **0.1** |
|  | **0.4** | **0.1** |
| Mean ± SD | **0.3 ± 0.09** | **0.1 ± 0.03** |
| **Filtek™ Supreme XTE** | **1.7** | **0.6** |
|  | **1.1** | **0.4** |
|  | **0.9** | **0.3** |
| Mean ± SD | **1.2 ± 0.41** | **0.4 ± 0.15** |
| **Tetric EvoFlow®** | **6.3** | **2.2** |
|  | **8.8** | **3.1** |
|  | **6.8** | **2.4** |
| Mean ± SD | **7.3 ± 1.29*** | **2.6 ± 0.46*** |
| **Clinpro™ Sealant** | **3.1** | **0.9** |
|  | **0.8** | **0.2** |
|  | **0.7** | **0.2** |
| Mean ± SD | **1.5 ± 1.35** | **0.5 ± 0.41** |
| **DELTON®** | **6.2** | **1.9** |
|  | **6.5** | **2.0** |
|  | **5.8** | **1.8** |
| Mean ± SD | **6.2 ± 0.33*** | **1.9 ± 0.10*** |
| **Helioseal® F** | **0.6** | **0.2** |
|  | **0.5** | **0.1** |
|  | **0.5** | **0.1** |
| Mean ± SD | **0.5 ± 0.10** | **0.2 ± 0.04** |
| **Transbond™ Plus** | **0.8** | **0.3** |
|  | **1.0** | **0.3** |
|  | **0.6** | **0.2** |
| Mean ± SD | **0.8 ± 0.22** | **0.3 ± 0.07** |
| **Band-Lok®** | **2.2** | **0.7** |
|  | **2.4** | **0.7** |
|  | **9.7** | **3.0** |
| Mean ± SD | **4.7 ± 4.3** | **1.5 ± 1.30** |

**Table S3. Study 1.** Total BPA leaching from cured materials after 2 weeks

| **Sample** | **BPA leaching [ng/ml]** | **BPA leaching [ng/cm^2^]** |
| --- | --- | --- |
| **Control** | **1,3** | **not appropriate** |
|  | **2,0** |  |
|  | **1.5** |  |
| Mean ± SD | **1.6 ± 0.36** |  |
| **ceram.X®** | **2.7** | **1.0** |
|  | **0.9** | **0.3** |
|  | **Lost** | **Lost** |
| Mean ± SD | **1.8 ± 1.23** | **0.6 ± 0.43** |
| **Grandio® Flow** | **0.3** | **0.1** |
|  | **0.2** | **0.1** |
|  | **0.4** | **0.1** |
| Mean ± SD | **0.3 ± 0.14** | **0.1 ± 0.05** |
| **Filtek™ Supreme XTE** | **0.7** | **0.2** |
|  | **1.3** | **0.5** |
|  | **1.0** | **0.4** |
| Mean ± SD | **1.0 ± 0.32** | **0.4 ± 0.11** |
| **Tetric EvoFlow®** | **7.2** | **2.6** |
|  | **8.4** | **3.0** |
|  | **10.4** | **3.7** |
| Mean ± SD | **8.7 ± 1.60** | **3.1 ± 0.56** |
| **Clinpro™ Sealant** | **0.8** | **0.2** |
|  | **0.8** | **0.2** |
|  | **1.2** | **0.4** |
| Mean ± SD | **0.9 ± 0.24** | **0.3 ± 0.07** |
| **DELTON®** | **8.6** | **2.6** |
|  | **11.6** | **3.5** |
|  | **7.3** | **2.2** |
| Mean ± SD | **9.2 ± 2.21** | **2.8 ± 0.67** |
| **Helioseal® F** | **1.3** | **0.4** |
|  | **0.3** | **0.1** |
|  | **0.3** | **0.1** |
| Mean ± SD | **0.6 ± 0.60** | **0.2 ± 0.18** |
| **Transbond™ Plus** | **0.6** | **0.2** |
|  | **1.7** | **0.5** |
|  | **0.5** | **0.1** |
| Mean ± SD | **0.9 ± 0.66** | **0.3 ± 0.20** |
| **Band-Lok®** | **3.2** | **1.0** |
|  | **3.3** | **1.0** |
|  | **6.0** | **1.8** |
| Mean ± SD | **4.2 ± 1.6** | **1.3 ± 0.48** |

**Table S4.** Complete data set, Study 2.

| **Material** |  | **BPA leaching [ng/ml]** | **BPA leaching [ng/cm^2^]** | **Material** |  | **BPA leaching [ng/ml]** | **BPA leaching [ng/cm^2^]** |
| --- | --- | --- | --- | --- | --- | --- | --- |
| **ceram.X®** | **1** | **0.36** | **0.13** | **Clinpro™ Sealant** | **1** | **0.28** | **0.10** |
|  | **2** | **0.37** | **0.13** |  | **2** | **0.25** | **0.09** |
|  | **3** | **0.29** | **0.10** |  | **3** | **0.11** | **0.04** |
|  | **4** | **0.45** | **0.16** |  | **4** | **0.13** | **0.05** |
|  | **5** | **0.35** | **0.12** |  | **5** | **0.09** | **0.03** |
| **Mean ± SD** | | **0.36 ± 0.06** | **0.13 ± 0.02** | **Mean ± SD** | | **0.17 ± 0.09** | **0.06± 0.03** |
| **Grandio® Flow** | **1** | **0.09** | **0.03** | **DELTON®** | **1** | **8.75** | **3.09** |
|  | **2** | **0.08** | **0.03** |  | **2** | **12.38** | **4.38** |
|  | **3** | **0.10** | **0.04** |  | **3** | **7.98** | **2.82** |
|  | **4** | **0.06** | **0.02** |  | **4** | **11.40** | **4.03** |
|  | **5** | **0.08** | **0.03** |  | **5** | **7.43** | **2.63** |
| **Mean ± SD** | | **0.08 ± 0.01** | **0.029 ± 0.005** | **Mean ± SD** | | **9.6 ± 2.2** | **3.4 ± 0.8** |
| **Filtek™ Supreme XTE** | **1** | **0.68** | **0.24** | **Helioseal® F** | **1** | **0.03** | **0.01** |
|  | **2** | **0.65** | **0.23** |  | **2** | **0.09** | **0.03** |
|  | **3** | **0.52** | **0.18** |  | **3** | **0.01** | **0.00** |
|  | **4** | **0.45** | **0.16** |  | **4** | **0.08** | **0.03** |
|  | **5** | **0.49** | **0.17** |  | **5** | **0.00** | **0.00** |
| **Mean ± SD** | | **0.6 ± 0.1** | **0.20 ± 0.04** | **Mean ± SD** | | **0.04 ± 0.04** | **0.01 ± 0.01** |
| **Tetric EvoFlow®** | **1** | **6.46** | **2.29** | **Transbond™ PLUS** | **1** | **0.12** | **0.04** |
|  | **2** | **6.07** | **2.15** |  | **2** | **0.09** | **0.03** |
|  | **3** | **7.23** | **2.56** |  | **3** | **0.10** | **0.04** |
|  | **4** | **6.67** | **2.36** |  | **4** | **0.08** | **0.03** |
|  | **5** | **6.29** | **2.22** |  | **5** | **0.08** | **0.03** |
| **Mean ± SD** | | **6.5 ± 0.4** | **2.3 ± 0.2** | **Mean ± SD** | | **0.09 ± 0.02** | **0.033 ± 0.006** |
| **Blank** | **1** | **0.01** | **not appropriate** | **Band-Lok®** | **1** | **1.49** | **0.53** |
|  | **2** | **0.00** |  |  | **2** | **0.94** | **0.33** |
|  | **3** | **0.00** |  |  | **3** | **2.42** | **0.86** |
| **Mean ± SD** | | **0.0 ± 0.01** |  |  | **4** | **1.65** | **0.58** |
|  | | | |  | **5** | **1.16** | **0.41** |
|  |  |  |  | **Mean ± SD** | | **1.5 ± 0.6** | **0.5 ± 0.2** |

***Estimate of exposure.* *Comparison of our data with ADA and Van Landuyt***

We do not have data from saliva, however if we assume that the highest BPA levels we measured in the deionized water after 24 h (9.6 ng/ml → 0.5 ml sample volume→ 4.8 ng leached amount. 4.8 ng, surface area of test material 141.4 mm^2^ → 0.034 ng/mm^2^). Thus, 0.034 ng mm^2^ is what an individual may be exposed to over 24 h. This we can compare with the total amount found by ADA (2016) and per surface area with Van Landuyt (2011).

The latter found a geometric mean BPA release of 0.01752 nmol/mm^2^ (0.018 nmol/mm^2^). Converted to amount per surface area, this equals approximately 4.1 ng/mm^2^, using the molecular weight of BPA, 228.29 g/mol.

If we estimate the surface of the sealer used per tooth to be approximately 6 mm^2^ (2 mm x 3 mm) and the use of sealer on 4 primary molars, the total surface area is 24 mm^2^ (0.24 cm^2^). If we, as an example, take the leached amount from the sealer with highest leaching in Study 2, this was 0.034 ng/mm^2^). Our estimate gives an exposure to BPA from sealant on 4 teeth (24 mm^2^) of 0.82 ng the first 24 h which is somewhat higher than the 0.09 ng reported by ADA (2016).

For a child weighing 25 kilo, our estimate gives a daily dose of approximately 0.027 ng/kg bodyweight per day. Compared to the t-TDI for BPA of 4000 ng/kg bodyweight per day, the contribution from the sealant is far below this. Also considering that the exposure to this low concentration of BPA from the sealant will be brief, the contribution of BPA from sealant to the total exposure is negligible.
